# Iron in airway macrophages and infective exacerbations of chronic obstructive pulmonary disease

**DOI:** 10.1186/s12931-022-01929-7

**Published:** 2022-01-12

**Authors:** Terence Ho, Matthew Nichols, Gayatri Nair, Katherine Radford, Melanie Kjarsgaard, Chynna Huang, Anurag Bhalla, Nicola Lavigne, Manali Mukherjee, Michael Surette, Joseph Macri, Parameswaran Nair

**Affiliations:** 1 Department of Medicine, McMaster University, Hamilton, Canada; 2 Firestone Institute for Respiratory Health, St. Joseph’s Healthcare Hamilton, 50 Charlton Avenue East, Hamilton, ON L8N 4A6 Canada; 3 Department of Pathology and Laboratory Medicine, Western University, London, Canada; 4 St. Joseph’s Healthcare Hamilton, Hamilton, Canada; 5 Department of Pathology and Molecular Medicine, McMaster University, Hamilton, Canada

**Keywords:** COPD, Exacerbation, Sputum, Infection, Iron

## Abstract

**Background:**

Excess pulmonary iron has been implicated in the pathogenesis of lung disease, including asthma and COPD. An association between higher iron content in sputum macrophages and infective exacerbations of COPD has previously been demonstrated.

**Objectives:**

To assess the mechanisms of pulmonary macrophage iron sequestration, test the effect of macrophage iron-loading on cellular immune function, and prospectively determine if sputum hemosiderin index can predict infectious exacerbations of COPD.

**Methods:**

Intra- and extracellular iron was measured in cell-line-derived and in freshly isolated sputum macrophages under various experimental conditions including treatment with exogenous IL-6 and hepcidin. Bacterial uptake and killing were compared in the presence or absence of iron-loading. A prospective cohort of COPD patients with defined sputum hemosiderin indices were monitored to determine the annual rate of severe infectious exacerbations.

**Results:**

Gene expression studies suggest that airway macrophages have the requisite apparatus of the hepcidin-ferroportin axis. IL-6 and hepcidin play roles in pulmonary iron sequestration, though IL-6 appears to exert its effect via a hepcidin-independent mechanism. Iron-loaded macrophages had reduced uptake of COPD-relevant organisms and were associated with higher growth rates. Infectious exacerbations were predicted by sputum hemosiderin index (β = 0.035, p = 0.035).

**Conclusions:**

We demonstrate in-vitro and population-level evidence that excess iron in pulmonary macrophages may contribute to recurrent airway infection in COPD. Specifically, IL-6-dependent iron sequestration by sputum macrophages may result in immune cell dysfunction and ultimately lead to increased frequency of infective exacerbation.

**Supplementary Information:**

The online version contains supplementary material available at 10.1186/s12931-022-01929-7.

## Background

Chronic obstructive pulmonary disease (COPD) is a respiratory condition characterized by partially reversible airflow limitation and frequent exacerbations [1]. Acute exacerbations of COPD (AECOPD) are amongst the most common reason for admission to hospital [2], with infections estimated to be the underlying etiology in 50–70% of cases [3, 4].

Iron is a ubiquitous element and an essential nutrient to nearly all organisms [5]. It is necessary for the generation of reactive oxygen species, which is crucial for cellular bactericidal capacity. As a growth essential nutrient, increased iron may create a favourable environment for bacterial growth and increased virulence [6]. Systemic iron regulation, which occurs primarily by cellular sequestration, has been well-described [7]. IL-6 acts on hepatocytes and macrophages to stimulate the release of hepcidin, which subsequently causes the degradation of membrane-bound ferroportin. As the only known exporter of iron, the degradation of ferroportin leads to iron being trapped intracellularly. In the respiratory tract, these mechanisms are poorly understood, with the current evidence almost exclusively from murine models [8, 9].

Chronic inflammation can lead to the over-accumulation of iron, best described in anemia or chronic disease in hemodialysis patients [10]. Excess iron has also been implicated in the development of chronic diseases such as atherosclerosis and Alzheimer’s disease [6]. Recently, excess pulmonary iron has been shown to potentially play an important role in the pathogenesis of pulmonary fibrosis [11] and asthma [12]. Iron accumulation also appears to be important in COPD, as demonstrated by correlations between lung iron content and airflow obstruction and emphysema severity [13], and between airway iron and exacerbation [14], as well as proteomic analyses and genome-wide association studies of genes of iron metabolism [15]. Murine experiments have demonstrated that chelation of free iron prevented cigarette smoke-induced emphysema in mice [15]. The mechanism by which iron could cause susceptibility to COPD is not clear, but could be related to bacterial colonisation and infection of the airways, as supported by cell-line studies of experimental iron-loading demonstrating lysosomal dysfunction and reduced bactericidal capacity [16], and improvement in bactericidal activity with chelation in murine alveolar macrophages [17]. Such experiments have not been performed with respiratory-specific human pathogens in mind, or in human airway macrophages.

We hypothesized that raised respiratory iron content in COPD will increase the susceptibility to respiratory tract infection and subsequently lead to recurrent exacerbation. Our specific objectives were to: (1) Assess the mechanisms of human pulmonary macrophage iron sequestration; (2) Test the effect of macrophage iron-loading on cellular immune function; and (3) Prospectively determine if sputum hemosiderin index can predict infectious exacerbations of COPD.

## Methods

### Objective 1: Mechanisms of human pulmonary macrophage iron sequestration

To address this objective, serum and sputum measurements from a clinical cohort of COPD patients (as described below), real time quantitative polymerase chain reaction (RT-qPCR) of sputum cells from select patients within the COPD cohort and healthy controls, and cell-line derived and sputum macrophage experiments from healthy controls, were pursued.

#### Clinical cohort for prospective study

Patients were recruited from a single-centre during an admission for AECOPD (primary diagnosis) from December 2016 to May 2018. Informed consent was obtained from all subjects. The study was approved by the Hamilton Integrated Research Ethics Board (Project #1281). Inclusion in the study required age ≥ 40 years, one or more features of COPD/emphysema (airflow obstruction [FEV_1_/FVC < 0.7], radiologic evidence of emphysema), in the setting of a ≥ 10 pack-year history of cigarette smoking. Those who required admission to the intensive care unit, were unable to produce a spontaneous sputum, or had a history of asthma or bronchiectasis of greater than mild severity radiographically [18], were excluded.

#### Serum and sputum measurements in clinical cohort

Serum and spontaneous or induced sputum, were collected during exacerbation and at least 8-weeks post-discharge (stable state). Sputum samples were processed as previously described [19] to yield cell-free supernatant and a cell pellet.

Iron content in the airways was measured at exacerbation and stable state by two methods: staining of sputum cells with Prussian Blue to yield a sputum macrophage hemosiderin index (SHI) and quantification of free iron in the sputum supernatant by inductively-coupled mass spectrometer (ICP-MS). For additional detail please refer to the see Additional File 1: Methods.

For planned measurement of fluid phase analytes in sputum, supernatants were supplemented with protease cocktail inhibitor (Roche™, Switzerland) and stored at −70 °C for detecting fluid-phase analytes. IL-6 and hepcidin (R&D Systems™, Minneapolis, MN) were measured in both serum and sputum supernatant samples by enzyme-linked immunosorbent assay (ELISA). Lower limits of detection were 9.38 pg/mL and 3.12 pg/mL, respectively. Both ELISAs were performed following the user’s manuals with substitution of Strepavidin alkaline phosphatase rather than the Strepavidin horseradish peroxidase provided in the kits [20].

#### RNA extraction and RT-qPCR

Messenger ribonucleic acid was isolated from frozen whole sputum cell pellets (previously stored at − 80 °C in 90% fetal calf serum [Invitrogen™, Carlsbad, CA] and 10% dimethyl sulfoxide freezing media [Sigma-Aldrich™, St. Louis, MO]) from 17 COPD subjects (9 with SHI < 10 and 8 with SHI ≥ 10), and 5 healthy controls (never smokers with no known respiratory or cardiac disease) by magnetic microbeads (µMACS™ mRNA isolation kit, Miltenyi™, Bergisch Gladbach, Germany), then reverse-transcribed to create complementary deoxyribonucleic acid (cDNA; µMACS™ cDNA kit, Miltenyi™, Bergisch Gladbach, Germany), and stored at -80 °C. Nanodrop™ One (ThermoFisher Scientific™, Waltham, MA) was used to quantify the concentration of cDNA present. The COPD patients that participated in this part of the study were recruited consecutively during study-related follow-up. Expression levels of human hepcidin antimicrobial peptide (HAMP) and ferroportin (SLC40A1) genes were reported relative to the GAPDH housekeeping gene. The primer sequences and additional methods are included in Additional file 1: Methods.

The difference in cycle threshold (ΔCt) for each gene of interest was calculated by subtracting Ct for GAPDH from that of the gene of interest (ΔCt = Ct_gene_ − Ct_GAPDH_). To determine the relative expression in COPD subjects, the ΔCt for the gene of interest in these subjects was compared to the average ΔCt in healthy controls by the following equation: 2^−(ΔCtpatient −ΔCthealthyaverage)^.

#### Macrophage iron sequestration experiments

THP1 cells were cultured, passaged, and differentiated into THP1-derived macrophages (TDM). TDMs were then sub-cultured with media containing FeSO_4_ 100 µM and one of the experimental conditions (listed below). For further details regarding TDM differentiation and validation of cellular iron sequestration in the setting of iron-enriched media, please see Additional file 1. The experimental conditions consisted of: recombinant human IL-6 at 50 ng/mL (Abcam™, Cambridge, UK), recombinant human hepcidin at 1 µg/mL (Peptides International™, Louisville, KY), heat-inactivated HI (1 × 10^6^ bacteria in 35 µL PBS; hi-HI), hi-HI with IL-6, hi-HI with hepcidin, IL-6 with hepcidin, and hi-HI with IL-6 and hepcidin. Heat inactivation of HI was achieved by incubating at 55 °C for 10 min. Cells were sub-cultured under these conditions for 24-h, after which the plate was centrifuged, and the supernatant aspirated and stored at -20 °C. After the supernatant was completely aspirated, each well was washed with 2 mL of PBS, and then incubated with 0.5 mL of 0.25% trypsin (Gibco™, Gaithersburg, MD) to lift the cells. The main outcome for the TDM experiments was the intracellular iron content as measured by ICP-MS.

These experiments were completed on sputum macrophages (SM) from healthy controls (never smokers with no known respiratory or cardiac disease), with similar methods. Sputum macrophages were isolated by plastic adherence from fresh sputum cell pellets (as previously described; [9]). Post-isolation samples that contained < 85% macrophages (on hematoxylin and eosin stained cytospin slides) were discarded. Differently than TDM experiments, SM experiments included only IL-6 and hepcidin as conditions, and utilized Accutase™ (Sigma-Aldrich™, St. Louis, MO) and cell-scraper to lift cells. Iron efflux, as determined by the difference in iron concentration of control media from that of the supernatant after 24-h of incubation (both measured by ICP-MS), was chosen as the main outcome for these SM experiments.

### Objective 2: The effect of iron-loading on macrophage immune function

As described below, bacterial killing assays for TDM and SM were pursued to examine this objective.

#### Bacterial killing assay for sputum macrophages

Isolated sputum macrophages (by plastic adherence) from 10 different COPD cohort patients who were recruited consecutively during a period of clinical stability were diluted to a standardized concentration with 1 × Hank’s Balanced Salt Solution and combined with *H. influenzae* strain to achieve a multiplicity of infection of 10 and then incubated on a shaker at 200 rpm. At 1-h and 1.5-h, a 100 µL aliquot was removed, centrifuged at 3000 rpm for 4.5 min and then resuspended with 100 µL of sterile 1 × Hank’s Balanced Salt Solution to remove extracellular bacteria. This solution was then serially diluted with sterile deionized water (10^–1^ to 10^–5^) to lyse the macrophages, and then 10µL plated in duplicate on the appropriate agar. Agar plates were left under the hood to dry (approximately 10 min), and then incubated overnight. The next day, colony-forming units (CFU) were counted after 24-h of incubation for each dilution where possible and CFU/mL calculated. Bacterial killing after 1.5-h incubation was determined by comparing the CFU/mL at this time point with the CFU/mL at 1-h.

#### Bacterial uptake and killing assays for THP1-derived macrophages

TDM were sub-cultured in control media (RPMI 1640 with L-glutamine [2 mM], 10% fetal calf serum and Phorbol 12-myristate-13-acetate of 100 nM) or iron-enriched media (supplemented with FeSO_4_ at 250 µM) for 48 h and then lifted as previously described. Viable TDMs were combined with bacteria (grown to OD_600_ of 0.5) at a multiplicity of infection of 10 and then incubated on a shaker at 200 rpm. The experiment then proceeded similar to SM bacterial uptake and killing assays, but including time points of 1-h, 1.5-h, 2-h, 3-h, and 5-h. The main outcome of this experiment was the bacterial uptake of iron-loaded TDM, which was determined by comparing the CFU/mL to the control TDM at the 1-h time point.

### Objective 3: Sputum hemosiderin index as a predictor of infectious AECOPD

The previously described prospective clinical cohort was utilized to address this objective.

#### Prospective COPD cohort

Participants in the prospective cohort were monitored for 1-year for severe (requiring hospital admission) infectious exacerbations (primary outcome). As mentioned above, airway iron indices (SHI and ICP-MS) were measured at the time of exacerbation and after discharge during a stable state (see Additional file 1: Methods for further detail). At the time of severe AECOPD, an underlying infectious etiology was investigated by respiratory virus polymerase chain reaction by nasopharyngeal swab (RV-PCR), and spontaneous sputum culture and differential and cell count. An infectious exacerbation was defined as ≥ 1 of: positive respiratory virus RV-PCR, positive sputum culture, and/or total sputum neutrophil count ≥ 12 million cells/g. AECOPD was confirmed by a change in sputum appearance, sputum volume, or increased dyspnea by the admitting service [21].

### Statistics

For the prospective cohort data, descriptive statistics are presented as mean ± SD for parametric data and median (IQR) for non-parametric data. Linear regression and Spearman correlations were used to determine the associations between variables. Comparisons between two groups were accomplished by t-test (parametric) or Mann–Whitney test (non-parametric). In instances where paired testing could have been applied but sputum data were missing, we chose to analyze in an unpaired fashion. Comparisons between multiple groups were performed by ANOVA (with Dunnett’s multiple comparisons test) for parametric data and Kruskal–Wallis tests (with Dunn’s multiple comparisons test) for non-parametric data. Group comparisons of grouped data were accomplished by Friedman test. p < 0.05 was considered statistically significant. All analyses were performed using either SPSS 23.00. IBM Corporation, Armonk, NY, USA, or GraphPad Prism version 8.0.0 for Mac OS X, GraphPad Software, San Diego, California USA, www.graphpad.com. Using previously reported annualized COPD exacerbation rates (μ = 1.53, σ = 1.05) [22], to detect an absolute difference in infective exacerbation frequency of 50%, we estimated that we would require 46 subjects total assuming a beta of 0.2 and alpha of 0.05 (one-sided test).

## Results

### Prospective clinical cohort

Fifty-six subjects consented to participate in the study, of which 49 completed the 1-year monitoring period. Seven subjects were lost to follow-up due to death (n = 4), being unreachable by telephone (n = 2), and withdrawing due to a new diagnosis of cancer (n = 1). Baseline characteristics are summarized in Table 1. Parameters related to systemic iron and echocardiogram results are summarized in Additional file 2: Table S1.

**Table 1:** Baseline characteristics of prospective clinical cohort

Demographics	Value
Number of participants	49
Age (years)	67 ± 9
Male/Female	32/17
Caucasian (%)	94
COPD-related variable	
LAMA use (n,%)	45 (91.8%)
Inhaled corticosteroid use (n,%)	43 (87.5%)
LABA use (n,%)	40 (81.6%)
Current smoker (%)	57.1
Smoking history (pack-years)	50 (39,63)
Radiographic emphysema (%)	90.6
Home O_2_ (%)	38.3
FEV_1_ (L)	1.1 ± 0.47
FEV_1_ (%Predicted)	41 ± 18
Self-reported AECOPD in last 1-year	2 (0,4)
Self-reported Admission for AECOPD in last 1-year	0 (0,1)

*LAMA* long-acting muscarinic antagonist,* FEV1* forced expiratory volume in 1-s,* AECOPD* acute exacerbation of chronic obstructive pulmonary disease

### Mechanisms of macrophage iron sequestration

**Fig. 1:**
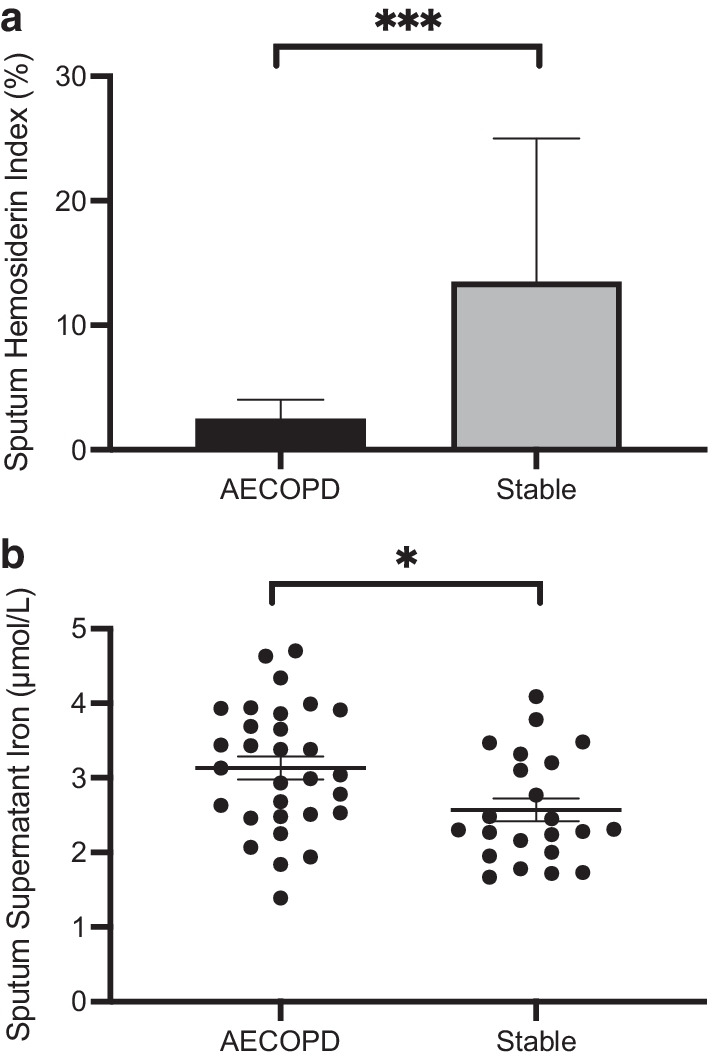
a Sputum hemosiderin index in subjects during acute exacerbation and clinical stability. ***p < 0.001. Data shown as median (IQR). *AECOPD* acute exacerbation of chronic obstructive pulmonary disease. **b** Sputum supernatant iron concentration in subjects during acute exacerbation and clinical stability. Data shown as mean ± SEM. *p < 0.05. *AECOPD* acute exacerbation of chronic obstructive pulmonary disease

Within the patient cohort, the sputum hemosiderin index collected during clinical stability was significantly higher than during AECOPD (median 14[4.3,31]% vs 2.5[1.0,8.8], p = 0.0001, Mann–Whitney test; Fig. 1a). The levels of unbound iron demonstrated the opposite with lower values during clinical stability than at AECOPD (2.6 ± 0.73 vs 3.1 ± 0.82 μM, p = 0.015, unpaired t-test; Fig. 1b; Table 2). Neither serum or sputum IL-6 changed significantly at AECOPD compared to follow-up. On the other hand, serum hepcidin was significantly higher during AECOPD than during follow-up while sputum hepcidin did not demonstrate such a difference (Summarized in Table 2). SHI at follow-up was correlated with sputum IL-6 (r = 0.37, p = 0.045, Spearman; Linear regression shown in see Additional file 3: Fig. S1a) and serum IL-6 (r = 0.31, p = 0.086, Spearman; Linear regression shown in see Additional file 4: Fig. S1b) measured during AECOPD. Neither SHI during AECOPD or during follow-up were correlated with serum or sputum hepcidin. To examine the contribution of cigarette smoking to sputum iron, SHI and supernatant iron levels were compared stratified based on smoker’s inclusions in the sputum (None, Few, Moderate, Many), and smoking status (current versus ex-smoker). The analysis of SHI was limited by only three patients with at least moderate smoker’s inclusions and a viable SHI at exacerbation. Otherwise, when post-AECOPD SHI or supernatant iron during AECOPD or post-AECOPD were stratified by smoker’s inclusions or smoking status, no associations were seen.

**Table 2:** Measures of airway intra- and extra-cellular iron, and potential iron-regulatory proteins

Serum/sputum variables	AECOPD	Post-AECOPD	p-value
Sputum hemosiderin index (%)	2.5 (1.0,8.8)	14.0 (4.3,31.0)	0.0001
Sputum free iron (μM)	3.1 ± 0.8	2.6 ± 0.7	0.015
Serum IL-6 (pg/mL)	2.8 (2.3,36.6)	3.4 (2.2,10.7)	0.94
Sputum IL-6 (pg/mL)	63.4 (11.5,211.0)	111.0 (34.2, 198.0)	0.19
Serum Hepcidin (ng/mL)	77.8 (18.9,111.4)	24.8 (4.6,79.6)	0.01
Sputum Hepcidin (pg/mL)	31.1 (8.0,71.8)	15.5 (6.2,29.1)	0.16

Measurements made during AECOPD and during a stable state at least 8-weeks after exacerbation. Data presented as mean ± SD or median (IQR)

*AECOPD* acute exacerbation of chronic obstructive pulmonary disease; *IL-6* interleukin-6

**Fig. 2:**
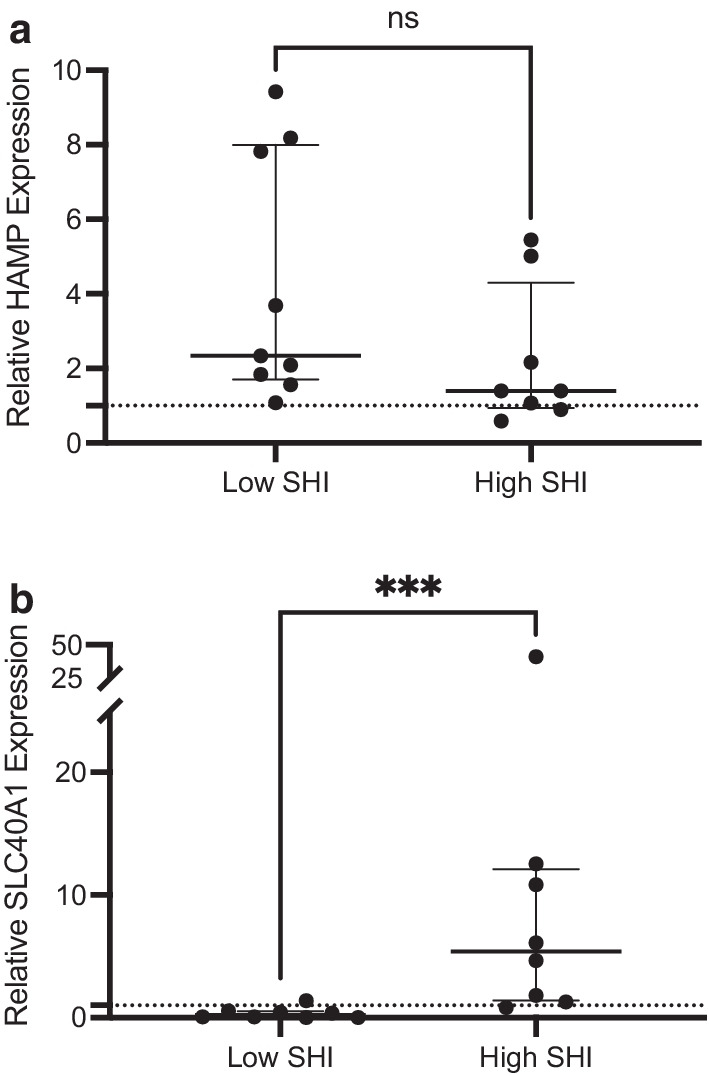
**a** Relative gene expression of hepcidin in COPD patients based on stable sputum macrophage iron content. Data shown as median (IQR). *HAMP* hepcidin antimicrobial peptide; *SHI* sputum hemosiderin index. **b** Relative gene expression of ferroportin in COPD patients based on stable sputum macrophage iron content. Data shown as median (IQR). SLC40A1, solute carrier family 40 member 1; *SHI* sputum hemosiderin index. ***p < 0.001

In sputum cells of COPD patients, HAMP expression was increased relative to healthy controls with no difference demonstrated between patients with high and low intracellular iron (1.40 [0.94,4.30] vs. 2.34 [1.70,8.00] fold difference, p = 0.09, Mann–Whitney test, Fig. 2a). On the other hand, relative to healthy controls, SLC40A1 expression in COPD patients was elevated in the high intracellular iron group and reduced in those with low intracellular iron, with significantly greater expression in the high versus low group (5.38 [1.42,12.10] vs. 0.22 [0.03,0.51], p = 0.0006, Mann–Whitney test, Fig. 2b). There were no differences in age or sex between healthy controls, COPD patients with low SHI, and COPD patients with high SHI. No differences in smoking status and pack-year exposure were noted between low and high SHI groups (See Additional file 5: Table S2).

**Fig. 3:**
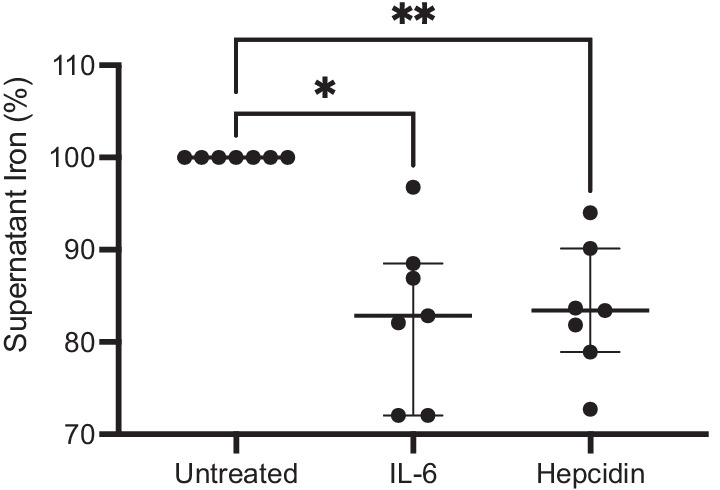
Reduction in supernatant iron for sputum macrophage experiments. N = 7. Data shown as median (IQR). *IL-6* interleukin-6. *p < 0.05; **p < 0.01

In experiments with SM from healthy controls (n = 7), there was a significant reduction in supernatant iron concentration when subculturing with IL-6 (median − 17.2 [− 28.0, − 11.5] %, p = 0.032) and hepcidin (median − 16.6 [− 21.1, − 9.9] %, p = 0.003, all by Dunn’s multiple comparisons test) compared with control, reflective of iron sequestration (Fig. 3). In TDM experiments (n = 4), sub-culturing in media with IL-6 (median 16.0 [12.4,18.0] μM, p = 0.014), hepcidin (median 17.2 [15.2,18.8] μM, p = 0.0028), and IL-6 with hepcidin (median 17.6 [9.71,20.9] μM, p = 0.014, all by Dunn’s multiple comparisons test; see Additional file 6: Fig. S2) led to increased intracellular iron content (as measured by ICP-MS) compared to control (median 2.00 [0.671,2.99] μM). Subculturing with IL-6 and hepcidin together led to a statistically significant increase in TDM hemosiderin index compared to control (data not shown, p < 0.05). IL-6, hepcidin, and hi-HI subculture resulted in a decrease in supernatant iron concentration compared to control (data not shown, p < 0.01).

### Macrophage iron and susceptibility to infection

In experiments with SM isolated from individuals from the COPD cohort, there was a near-significant correlation between SHI and the growth rate of *H.influenzae* at 0.5 h (r = 0.60, p = 0.075, Spearman correlation; see Additional file 7: Fig. S3). As an exploratory condition, three participants had bacterial growth measured in the presence of the chelator Deferoxamine at 100 nM (Abcam™, Cambridge, UK), which demonstrated reduced growth of *H.influenzae* compared to control (median − 128 [− 237, − 96]%, p = 0.25, Wilcoxon matched-pairs signed rank test; data not shown).

TDMs loaded with iron demonstrated a significant reduction in bacterial uptake compared to untreated cells for both *H.influenzae* (median − 28.3 [− 37.9, − 15.5] %, p = 0.002, Mann–Whitney test) and *S.pneumoniae* (median − 32.5 [− 50.6, − 12.0] %, p = 0.0006, Mann–Whitney test; data not shown). Evaluation of further time-points did not demonstrate differences in bactericidal capacity.

### Sputum hemosiderin index as a predictor of infectious AECOPD

Amongst index admissions, 32 were secondary to infection while 17 were non-infectious (criteria as defined in Methods). Infective exacerbations were confirmed by bacterial culture in 10 (31%), by RV-PCR (without positive bacterial culture) in 10 (31%), and by differential and cell count alone in 12 (38%). There were 73 readmissions for AECOPD during the follow-up period. Analysing by patient revealed that there was a median of 1(0,2), or mean of 1.49 ± 1.56 readmissions. A corresponding sputum sample and RV-PCR were collected in 40 (55%) and 52 (71.2%) of these episodes, respectively, of which 33 (45.2%) were proven to be infective exacerbations. For readmissions, infective exacerbation were confirmed by bacterial culture in 15 (45.5%), by RV-PCR (without positive bacterial culture) in 5 (15.1%), and by differential and cell count alone in 13 (39.4%).

Negative binomial regression was performed for SHI at AECOPD and during a clinically stable period, accounting for covariates of FEV_1_%Predicted, the number of admissions for AECOPD in the last 1-year, age, sex, and inhaled corticosteroid use. SHI while clinically stable was a significant predictor of infective exacerbations (β = 0.035, p = 0.035), and FEV_1_%Predicted was borderline significant (β = − 0.049, p = 0.051; Table 3). When serum IL-6 was added to the model, SHI was borderline predictive (β = 0.038, p = 0.05; see Additional file 8: Table S3).

**Table 3:** Negative binomial regression model

Coefficients	Estimate	Standard Error	p-value	95% CI	Rate ratio	95% CI
Intercept	0.115	2.603	0.965	− 4.987 to 5.217	1.122	0.007 to 184.361
Admissions in last year	0.098	0.213	0.646	− 0.320 to 0.516	1.103	0.726 to 1.675
FEV_1_%Predicted	− 0.049	0.025	0.051	− 0.099 to 0.000	0.952	0.906 to 1.000
SHI at follow-up	0.035	0.016	0.035	0.002 to 0.067	1.035	1.002 to 1.069
Age	− 0.008	0.045	0.860	− 0.097 to 0.081	0.992	0.908 to 1.084
Sex	0.417	0.620	0.502	− 0.799 to 1.633	1.517	0.450 to 5.119
ICS use	0.862	1.377	0.531	− 1.837 to 3.561	2.368	0.159 to 35.187

*FEV*_*1*_ forced expiratory volume in 1-s,* SHI* sputum hemosiderin index,* ICS* inhaled corticosteroid

## Discussion

In this study, experiments investigating the mechanisms of pulmonary iron sequestration and hypothesized macrophage dysfunction were paired with a prospective COPD cohort to determine if there is a coherent pathophysiologic mechanism by which increased sputum macrophage iron could predispose to infective AECOPD.

Regarding the mechanisms of pulmonary macrophage iron sequestration, there appears to be a role for both IL-6 and hepcidin. In contrast to tissue macrophages, we demonstrated that IL-6 caused pulmonary iron sequestration independent of hepcidin. This role for IL-6 was corroborated by the prospective cohort. Though gene expression studies provide evidence that the hepcidin-ferroportin axis is available within sputum macrophages, high iron levels within these cells in the presence of increased ferroportin gene expression would suggest that hepcidin plays a role in iron sequestration, but that this pathway is compensatory, rather than the main driver. Cigarette smoke is considered a source of pulmonary iron, but we were not able to demonstrate a consistent association between cigarette smoke and sputum iron levels. The only other study examining pulmonary iron sequestration focused on airway epithelial cells and performed limited macrophage experiments (where only hepcidin was studied). Contrary to our findings, they found that exogeneous hepcidin did not cause iron sequestration in pulmonary macrophages [23], which may be related to our experiments using a higher concentration of hepcidin corresponding with systemic inflammation. The roles of IL-6 and ferroportin on pulmonary macrophage iron sequestration have not been previously been studied in human cells.

In-vitro experiments of TDM and SM from COPD patients were used to test the effect of macrophage iron-loading on cellular immune function. Iron-loaded SM demonstrated increased bacterial growth rates. Due to low cell yield, an issue inherent when working with SM isolated by plastic adherence, as well as a lack of a real-time iron quantification required to have an independent control group, we were not able to determine if this increased growth rate in SM was related to an impairment of bacterial uptake or killing. However, the reduced bacterial uptake seen in TDM experiments suggests that this mechanism is more prominent. Other studies examining excess iron in macrophages are limited to cell-line or murine macrophages, and experimental conditions manipulating hepcidin rather than iron itself [24, 25]. A single previous study demonstrated a reduction in cell-line macrophage bacterial killing rate [16]. To our knowledge, this is the first study to assess the effect of iron content on human pulmonary macrophage immune dysfunction.

Finally, with a prospective COPD cohort, we demonstrated that sputum macrophage iron content shortly after exacerbation is a predictor of future infective AECOPD. Pulmonary iron varied between patients and clinical status (exacerbation versus stable), but did not appear to be related to smoking status. During AECOPD, free sputum iron increased, and after the exacerbation resolved, free sputum iron decreased with a concomitant increase in SHI, suggestive of active pulmonary macrophage iron sequestration. It is plausible that in those individuals with an increase in IL-6 associated with AECOPD, the iron sequestration process initiates and ultimately leads to elevated SHI detected after the exacerbation event. Our findings would suggest that elevated SM iron in these individuals impairs macrophage immune function and ultimately predisposes to infective AECOPD. Our group had previously described that SHI was predictive of infective AECOPD [26]. Despite that previous study being retrospective and using a less robust definition of infective exacerbation (based on self-reported antibiotic use), there was a similar rate ratio for SHI (for every 1% increment, there was a corresponding 3.5% increase in infective exacerbations). A recent observational study demonstrated higher bronchoalveolar lavage fluid iron in those who subsequently developed exacerbation compared those who did not [14]. Our study differed in that it examined patients with more severe COPD, and measured our outcome prospectively rather than retrospectively.

The first limitation of this study relates to a limited sample size of various sputum macrophage experiments, and a yield of sputum macrophages in the experiments that prevented confirmation of intracellular iron accumulation and gene expression studies. Unfortunately, this was unavoidable due to the nature of the macrophage isolation techniques. We feel that coupling this data with data from a prospective COPD cohort presented a convergent framework of pulmonary iron sequestration and predisposition to infection. There were some inconsistent findings between macrophage experiments and the prospective cohort. While IL-6 led to iron sequestration in macrophage experiments, sputum IL-6 was not significantly higher after exacerbation. However, we were able to demonstrate a correlation between SHI and sputum IL-6 (Fig. S1a), and there was also a trend towards higher sputum IL-6 post-AECOPD. Secondly, gene expression studies were conducted on a heterogeneous population of sputum cells rather than macrophages alone. However, all samples contained 1–3 million macrophages per gram of sputum, and aside from HAMP being expressed in neutrophils [27], neither HAMP or SLC40A1 are known to be expressed in other cell types found in sputum. Thirdly, exacerbations of moderate severity, that is not requiring emergency room visit or hospitalization, were not measured and certainly could have been of infectious etiology. However, we chose to focus on severe exacerbations as they are more reliably captured (as opposed to self-report), and it was more feasible to characterize these episodes by collecting adequate sputum samples in this context. Using this approach, we were able to accurately characterize participants’ exacerbations with RV-PCR and sputum cytometry, rather than rely only on a history of antibiotic use. Of note, the rate of hospitalizations due to COPD was high as compared to large population-based studies, highlighting that our cohort was particularly prone to exacerbations. Lastly, our focus was on macrophage iron sequestration, and thus we did not measure iron bound to iron-binding proteins such as ferritin or lipocalin-2.

In summary, this study demonstrates that excess airway macrophage iron occurs in a subset of patients with COPD, and when present prospectively predicts infectious exacerbation. Furthermore, we elucidate a plausible mechanism by which this may occur involving IL-6, and iron-related immune dysfunction of macrophages. Further research is required to fully understand the mechanisms of pulmonary iron sequestration in health and disease, and to determine if airway iron could be a target for therapeutic intervention.

## Supplementary Information

**Additional file 1: Supplementary Methods.** A supplement to the Methods section.

**Additional file 2: Table S1.** Systemic iron and echocardiographic parameters of COPD clinical cohort.

**Additional file 3: Figure S1a.** Linear regression of sputum supernatant interleukin-6 (by ELISA) and sputum hemosiderin index.

**Additional file 4: Figure S1b.** Linear regression of serum interleukin-6 (by ELISA) and sputum hemosiderin index.

**Additional file 5: Table S2:** Characteristics of RT-qPCR cohorts.

**Additional file 6: Figure S2.** Intracellular iron content of cell-line derived macrophages after 24-hour incubation with various conditions.

**Additional file 7: Figure S3.** Linear regression of sputum hemosiderin index and growth rate of *H. influenzae*.

**Additional file 8: Table S3:** Negative binomial regression model.

## Data Availability

The datasets used and/or analysed during the current study are available from the corresponding author on reasonable request.

## Abbreviations

ΔCt: Difference in cycle threshold
AECOPD: Acute exacerbation of chronic obstructive pulmonary disease
cDNA: Complementary deoxyribonucleic acid
CFU: Colony forming unit
COPD: Chronic obstructive pulmonary disease
ELISA: Enzyme linked immunosorbent assay
FEV1: Forced expiratory volume in 1 s
FVC: Forced vital capacity
HAMP: Hepcidin antimicrobial peptide
hi-HI: Heat-inactivated Haemophilus influenzae
ICP-MS: Inductively-coupled mass spectrometry
IL-6: Interleukin-6
RT-qPCR: Real-time quantitative polymerase chain reaction
RV-PCR: Respiratory virus polymerase chain reaction
SHI: Sputum hemosiderin index
TDM: THP1-derived macrophages
THP1: Tamm Horsfall Protein 1
