# Supplementary material for: Iron in airway macrophages and infective exacerbations of chronic obstructive pulmonary disease
Source: Respir Res. 2022 Jan 12;23:8. doi: 10.1186/s12931-022-01929-7 (PMC8756761; doi:10.1186/s12931-022-01929-7)
Supplement: Supplementary file 1 — Additional file 1: Supplementary Methods. A supplement to the Methods section. [file 12931_2022_1929_MOESM1_ESM.docx]

**Supplementary Methods**

Quantification of Iron

Iron concentrations were analyzed on an Agilent 8800 triple quadrupole inductively-coupled mass spectrometer (ICP-MS)™ (Santa Clara, CA) connected to an autosampler with subsequent data analysis in Masshunter software. Optimization of the ICP-MS was performed using an Agilent supplied autotuning solution which contained: Li, Y, Ce, TI, and Co at 10 PPM in a matrix of 2% HNO_3_. Concentrations were determined against a 13-point standard curve ranging from 0.002 µM to 4 µM.  Germanium and indium were both used as internal standards and acceptable recovery rates were set at 80-120%. Iron was analyzed using kinetic energy discrimination using high purity helium as the discriminatory gas with corresponding iron measurements at m/z 56 🡪 56 at both Q 1 and 3, respectively. The dwell time was set at 0.1 seconds with 25 sweeps/replicates for 3 replicates. All samples were diluted 25 or 50-fold prior to analysis into 0.1% ICP-MS grade nitric acid, 0.05 % triton X-100, 0.1% EDTA and 1% isopropanol prior to measurement. The diluent also contained the internal standards at a concentration of 10 ug/L.

Protein estimation (Protein Assay Kit II, Bio-rad™, Hercules, CA) was used to standardize the ICP-MS protocol for cell pellets after lysis with RIPA buffer (Sigma-Aldrich™, St. Louis, MO). Iron content was also measured semi-quantitatively with the macrophage hemosiderin index. It is determined by calculating the proportion of positively-staining macrophages with Perl’s Prussian Blue stain from a representative sampling of 100 macrophages under light microscopy as previously described {Leigh:1999fy}.

RNA Extraction and RT-qPCR

The primer sequences were as follows: human hepcidin: CTGCTGCGGCTGCTGTCATCGATCAAAGTGTGGGATGTGCTGCAAGACGTAGAACCTACCTGCCCTGCCCCCGTCCCCTCCCTTCCTTATTTATTCCTGCTGCCCCAGAACATAGGTCT; human ferroportin: GTAGGAGACCCATCCATCTCGGAAGGTACGGAAGGGCTCAGC CATCTGGGAGGCACAAGTAGGCTCTTGCTCATGTTCAAGCTCATGGATGTTAGAGTCTTTCACACCCATTAG; human TMPRSS6: CCAAAGGAATAGACGGAGCTGGAGTTGTA GTAAGTTCCCAGGCGGGTGCTGGTGATGAGCTCCTTGAGCATCTTCTGGGCTTTGGCGGTTTCACTGCGGAAGGCACTAGATTCCCGGCGGGTAAGATCCTGGGAGAAGTGGCGATTGAGTACACGCAGACTGCCTGAGTACACCTGG; and human GAPDH: GTATGACAA CGAATTTGGCTACAGCAACAGGGTGGTGGACCTCATGGCCCACATGGCCTCCAAGGAGTAAGACCCCTGGACCACCAGCCCCAGCAAGAGCACAAGAGGAAGAGAGAGACCCTCACTGCTGGGGAGTCCCTGCCACAC. Synthetic templates for the genes of interest were used as positive controls. All primers and synthetic templates were purchased from Bio-rad™ (Hercules, CA).

A QuantStudio3™ (ThermoFisher Scientific™, Waltham, MA) machine and its corresponding software were used to complete the reactions as per the suggested protocol (as per kit instructions) and analyse the results. Each sample was plated in triplicate. Briefly, in each well (of a 96-well plate), 3μL of sample cDNA was added to 1μL of primer, 10μL of SYBR green supermix (Bio-rad™, Hercules, CA), and 6μL of nuclease-free water. The cycling protocol involved an activation step (95ºC for 2 minutes) and then forty cycles of denaturation

Cell Culture

THP-1 cells were maintained in RPMI 1640 (Gibco™, Gaithersburg, MD) supplemented with L-glutamine (2mM) with 10% fetal calf serum and incubated at 37°C with 5% CO_2_. They were passaged weekly to keep the cellular concentration <1x10^6^ cells/mL. Cells were differentiated into THP-1-derived macrophages (TDM) at a concentration of 2x10^5^ cells/mL by culturing with the above media with 100nM Phorbol 12-myristate-13-acetate for 48 hours.

Validation of Iron Sequestration by Iron-Enriched Media

Loading of macrophages was accomplished by subculturing with media containing FeSO_4_ (ThermoFisher Scientific™, Waltham, MA) for 24-hours at concentrations of 0, 100, 250 or 500µM. The iron-loading protocol was validated in TDM with both inductively-coupled plasma mass spectrometry of the cell pellet (Figure A) and the hemosiderin index (Figure B).

Figure A: Cell-line-derived macrophage hemosiderin index after incubation with iron-supplemented cell-culture media. Experiments performed in duplicate (n=3).

Figure B: Iron concentration in cell-line-derived macrophages by mass spectrometry after 24-hour incubation with iron-supplemented cell-culture media. Experiments performed in duplicate (n=3).
