# Supplementary material for: Iron in airway macrophages and infective exacerbations of chronic obstructive pulmonary disease
Source: Respir Res. 2022 Jan 12;23:8. doi: 10.1186/s12931-022-01929-7 (PMC8756761; doi:10.1186/s12931-022-01929-7)
Supplement: Supplementary file 2 — Additional file 2: Table S1. Systemic iron and echocardiographic parameters of COPD clinical cohort. [file 12931_2022_1929_MOESM2_ESM.docx]

**Table S1: Systemic iron and echocardiographic parameters of COPD clinical cohort.**

| **Iron Parameters** | **Value** |
| --- | --- |
| Hemoglobin (g/L) | 131 ± 16.4 |
| Mean cell volume (fL) | 89.0 ± 10.5 |
| Ferritin (𝜇g/L) | 112 (31.0, 186) |
| Iron (𝜇mol/L) | 12.0 (9.00, 15.0) |
| Transferrin (g/L) | 2.71 ± 0.511 |
| Anemia (n,%) | 17 (35) |
| Microcytosis (n,%) | 8 (17) |
| Possible iron deficiency (n,%) | 8 (19.5) |
| Possible iron overload (n,%) | 6 (14.6) |
| Iron < lower limit of normal (n,%) | 36 (87.5) |
| Anemia of chronic disease (n,%) | 1 (2.4) |
| **Echocardiogram Interpretation** |  |
| Systolic dysfunction | 3 (7.5) |
| Diastolic dysfunction | 11 (27.5) |
| Systolic and diastolic dysfunction | 2 (5) |
| Significant valvular disease | 1 (2.5) |
