# Supplementary figures and images for: Iron in airway macrophages and infective exacerbations of chronic obstructive pulmonary disease
Source: Respir Res. 2022 Jan 12;23:8. doi: 10.1186/s12931-022-01929-7 (PMC8756761; doi:10.1186/s12931-022-01929-7)

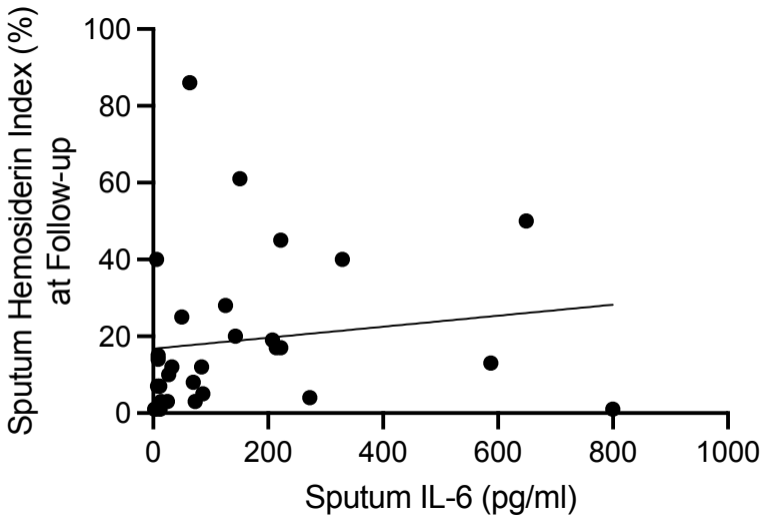

Supplement: Supplementary file 3 — Additional file 3: Figure S1a. Linear regression of sputum supernatant interleukin-6 (by ELISA) and sputum hemosiderin index. [file 12931_2022_1929_MOESM3_ESM.pdf]

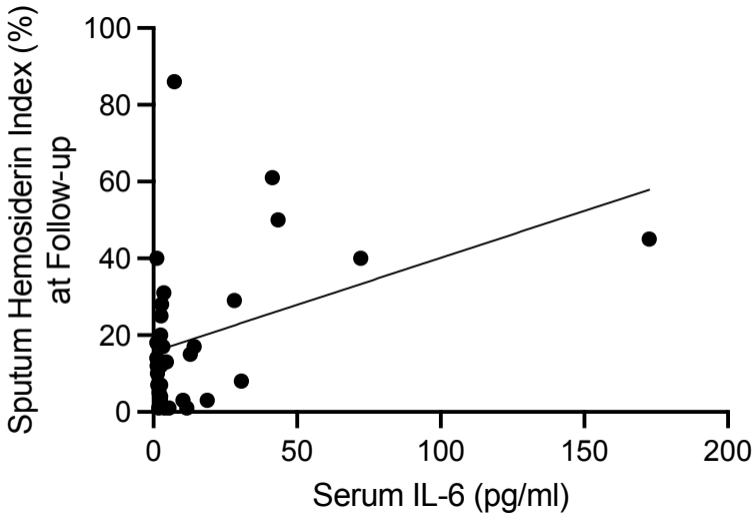

Supplement: Supplementary file 4 — Additional file 4: Figure S1b. Linear regression of serum interleukin-6 (by ELISA) and sputum hemosiderin index. [file 12931_2022_1929_MOESM4_ESM.pdf]

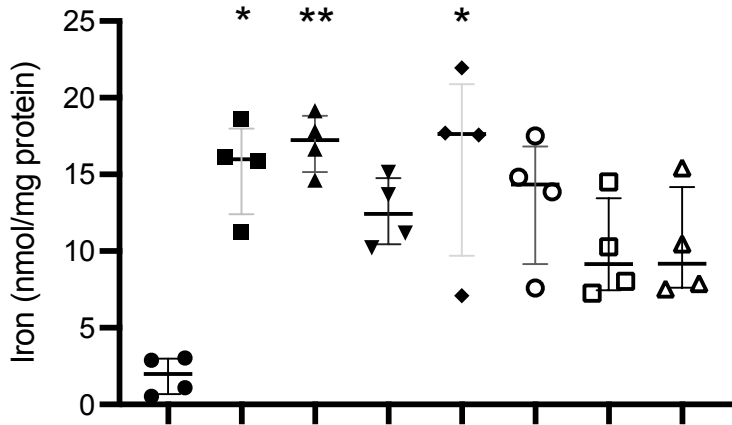

|                         |   |   |   |   |   |   |   |   |
|-------------------------|---|---|---|---|---|---|---|---|
| FeSO <sub>4</sub> 100μM | + | + | + | + | + | + | + | + |
| IL-6 50ng/mL            | - | + | - | - | + | + | - | + |
| Hepcidin 1μg/mL         | - | - | + | - | + | - | + | + |
| hi-Haemophilus          | - | - | - | + | - | + | + | + |

Supplement: Supplementary file 6 — Additional file 6: Figure S2. Intracellular iron content of cell-line derived macrophages after 24-hour incubation with various conditions. [file 12931_2022_1929_MOESM6_ESM.pdf]

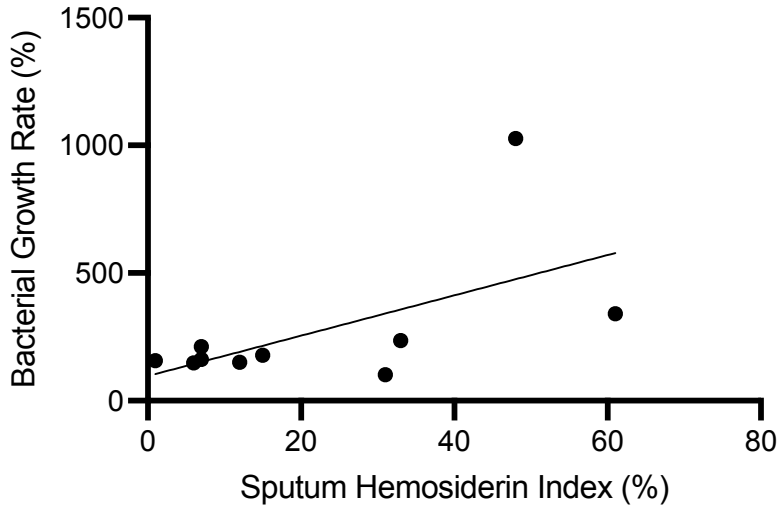

Supplement: Supplementary file 7 — Additional file 7: Figure S3. Linear regression of sputum hemosiderin index and growth rate of H. influenzae. [file 12931_2022_1929_MOESM7_ESM.pdf]
