# Supplementary material for: Iron in airway macrophages and infective exacerbations of chronic obstructive pulmonary disease
Source: Respir Res. 2022 Jan 12;23:8. doi: 10.1186/s12931-022-01929-7 (PMC8756761; doi:10.1186/s12931-022-01929-7)
Supplement: Supplementary file 5 — Additional file 5: Table S2: Characteristics of RT-qPCR cohorts. [file 12931_2022_1929_MOESM5_ESM.docx]

**Table S2: Characteristics of RT-qPCR cohorts**

| **Characteristic** | **Healthy Controls** | **Low SHI** | **High SHI** | **p-value** |
| --- | --- | --- | --- | --- |
| Age | 50 (33.5,65) | 70 (60,721.5) | 62.5 (58,71.5) | 0.13 |
| Female | 3 (60%) | 4 (44%) | 3 (37.5%) | 0.73 |
| Current smoker | 0 (0%) | 3 (33%) | 3 (37.5%) | 0.85 |
| Pack-years | N/A | 41 (30,59) | 50 (50,75) | 0.18 |

Data presented as Median (IQR) or Count (%). Kruskal-Wallis test performed for non-parametric data. Comparison of proportions by Chi-Square test.
