# Supplementary material for: Iron in airway macrophages and infective exacerbations of chronic obstructive pulmonary disease
Source: Respir Res. 2022 Jan 12;23:8. doi: 10.1186/s12931-022-01929-7 (PMC8756761; doi:10.1186/s12931-022-01929-7)
Supplement: Supplementary file 8 — Additional file 8: Table S3: Negative binomial regression model. [file 12931_2022_1929_MOESM8_ESM.docx]

Table S3: Negative binomial regression model.

| Coefficients | Estimate | Standard Error | P-value | 95% CI | Rate ratio | 95% CI |
| --- | --- | --- | --- | --- | --- | --- |
| Intercept | 0.359 | 3.063 | 0.907 | -5.644 to 6.361 | 1.431 | 0.004 to 578.818 |
| Admissions in last year | -0.009 | 0.236 | 0.971 | -0.470 to 0.453 | 0.991 | 0.625 to 1.573 |
| FEV_1_ %Predicted | -0.047 | 0.027 | 0.078 | -0.100 to  0.005 | 0.954 | 0.905 to 1.005 |
| SHI at follow-up | 0.038 | 0.019 | 0.050 | 0.000 to 0.076 | 1.039 | 1.000 to 1.079 |
| Age | -0.012 | 0.055 | 0.825 | -0.119 to 0.095 | 0.988 | 0.888 to 1.100 |
| Sex | 0.908 | 0.829 | 0.273 | -0.716 to 2.532 | 2.480 | 0.489 to 12.580 |
| ICS use | 0.824 | 1.542 | 0.593 | -2.198 to 3.846 | 2.280 | 0.111 to 46.817 |
| Serum IL-6 | -0.039 | 0.0255 | 0.129 | -0.089 to 0.011 | 0.962 | 0.915 to 1.011 |

FEV_1_, forced expiratory volume in 1-second; SHI, sputum hemosiderin index; ICS inhaled corticosteroid; IL-6 interleukin 6.
